# Pan-neurexin perturbation results in compromised synapse stability and a reduction in readily releasable synaptic vesicle pool size

**DOI:** 10.1038/srep42920

**Published:** 2017-02-21

**Authors:** Dylan P. Quinn, Annette Kolar, Michael Wigerius, Rachel N. Gomm-Kolisko, Hanine Atwi, James P. Fawcett, Stefan R. Krueger

**Affiliations:** 1 Department of Physiology & Biophysics, Dalhousie University, Halifax Nova Scotia B3H 1X6, Canada; 2 Department of Neuroscience Institute, Dalhousie University, Halifax Nova Scotia B3H 1X6, Canada; 3 Department of Pharmacology, Dalhousie University, Halifax Nova Scotia B3H 1X6, Canada; 4 Department of Psychology and Neuroscience, Dalhousie University, Halifax Nova Scotia B3H 1X6, Canada; 5 Department of Biology, Dalhousie University, Halifax Nova Scotia B3H 1X6, Canada

## Abstract

Neurexins are a diverse family of cell adhesion molecules that localize to presynaptic specializations of CNS neurons. Heterologous expression of neurexins in non-neuronal cells leads to the recruitment of postsynaptic proteins in contacting dendrites of co-cultured neurons, implicating neurexins in synapse formation. However, isoform-specific knockouts of either all α- or all β-neurexins show defects in synaptic transmission but an unaltered density of glutamatergic synapses, a finding that argues against an essential function of neurexins in synaptogenesis. To address the role of neurexin in synapse formation and function, we disrupted the function of all α- and β-neurexins in cultured hippocampal neurons by shRNA knockdown or by overexpressing a neurexin mutant that is unable to bind to postsynaptic neurexin ligands. We show that neurexin perturbation results in an attenuation of neurotransmitter release that is in large part due to a reduction in the number of readily releasable synaptic vesicles. We also find that neurexin perturbation fails to alter the ability of neurons to form synapses, but rather leads to more frequent synapse elimination. These experiments suggest that neurexins are dispensable for the formation of initial synaptic contacts, but play an essential role in the stabilization and functional maturation of synapses.

Neuronal circuit development is an intricate process that culminates in the maturation of specific synaptic contacts between functionally diverse pre- and postsynaptic neurons. The mature state is preceded by a highly dynamic stage of synapse refinement during which new synaptic contacts are formed at an increased rate while inappropriate synapses are weakened and eliminated. This developmental program fine-tunes neuronal circuits in an activity-dependent manner^1^,^2^,^3^. Cell adhesion proteins provide trans-synaptic contacts and are well suited to mediate synapse formation and plasticity^4^,^5^,^6^,^7^,^8^. Furthermore, mutations in genes encoding synaptic adhesion proteins have been linked to neurodevelopmental disorders^9^,^10^,^11^,^12^,^13^,^14^, suggesting a role of these gene products in synaptic refinement.

Neurexins (Nrxns) represent one family of structurally diverse presynaptic cell adhesion molecules that have been implicated in the structural and functional development of synapses. Nrxns bind transsynaptically to postsynaptic adhesion molecules of the neuroligin and LRRTM families as well as to GluR∆2/cerebellin and calsyntenin-3^15^,^16^,^17^,^18^,^19^. They are expressed from three different genes (Nrxn1, Nrxn2, and Nrxn3) employing two promoters in each gene^20^. The resulting longer α-Nrxn and the shorter β-Nrxn differ in their binding affinities for postsynaptic ligands, which is further modulated by alternative splicing^19^,^21^).

Initial studies indicated that Nrxns and Nrxn ligands have an important role in the formation of synapses by transsynaptically recruiting proteins to pre- and postsynaptic compartments. Thus, heterologous expression of Nrxn in non-neuronal cells elicited the accumulation of postsynaptic density proteins in dendrites at contact sites^22^. Similarly, expression of Nrxn ligands in non-neuronal cells resulted in the clustering of presynaptic active zone material within contacting axons^16^,^18^,^19^,^23^. Investigations into the function of Nrxns have also been aided by knockout studies in which specific Nrxn isoforms or Nrxns ligands were targeted. Deletion of either all 3 α-Nrxns^24^ or all 3 β-Nrxns^25^ resulted in an impairment of neurotransmission at glutamatergic synapses that was at least partly due to a reduction in neurotransmitter release. Surprisingly, however, the density of glutamatergic synapses in both α- and β-Nrxn-deficient mouse models was found to be unchanged^24^,^25^. Similarly, deletion or knockdown of individual or multiple postsynaptic Nrxn ligands resulted in attenuated neurotransmission, but failed to affect synapse density 26,27 (but see^28^). Collectively, these findings conflict with a proposed role of Nrxns in synaptogenesis. It is important to note, however, that in the aforementioned studies, transsynaptic adhesion between Nrxns and their postsynaptic ligands was only partially disrupted. In the mammalian CNS, Nrxns and Nrxn ligands are expressed in a highly redundant, albeit cell type-specific, manner in individual neurons^19^,^26^,^29^,^30^,^31^. It therefore remains possible that the remaining isoforms in these mouse models were fully sufficient to support a permissive function of Nrxns in synaptogenesis.

In this study, we address the role of Nrxns in synapse formation by interfering with the function of all Nrxn isoforms using two independent approaches. Our data reveal that a pan-Nrxn perturbation has profound consequences for the stabilization and functional maturation of synaptic contacts.

## Results

### Molecular tools for disrupting the function of all Nrxn isoforms

**Figure 1:**
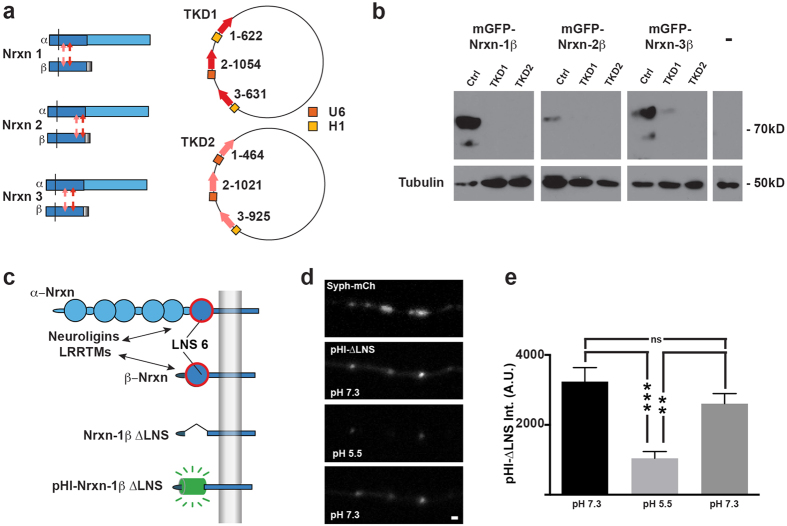
Molecular tools for disrupting Nrxn function. (**a**) For each of the 3 Nrxn gene transcripts, shRNA knockdown constructs were designed to target sequences present in both α and β Nrxn mRNA. Two different shRNA sequences were validated for each of the 3 Nrxn genes (pink and red arrows). shRNA sequences were combined into 2 unique Nrxn triple knockdown vectors (TKD1, TKD2). shRNA sequences for each TKD were driven by a combination of U6 and H1 promoters. (**b**) Knockdown efficiency was assessed by co-transfecting mGFP tagged versions of Nrxn 1β, 2β, and 3β along with Ctrl, TKD1 or TKD2 plasmids into HEK293 cells and performing a western blot with and an anti-GFP antibody. (**c**) A dominant negative Nrxn-1β construct was created by excising the extracellular LNS 6 domain of Nrxn-1β. The LNS 6 domain is essential for the binding of Nrxn with postsynaptic Neuroligins and LRRTMS. Nrxn-1β∆LNS reduces Nrxn-mediated transsynaptic cell adhesion by competing with endogenous α- and β-Nrxns for binding presynaptic scaffolding proteins. We analyzed the cellular properties of Nrxn-1β∆LNS by tagging it to a pH sensitive fluorescent molecule called pHluorin to create pHl- Nrxn-1β∆LNS. (**d**) Representative image of pHl-Nrxn-1β∆LNS (pHl-∆LNS) construct co-transfected with Synaptophysin-mCherry (Syph-mCh). Co-localization of pHl-∆LNS with Syph-mCh suggests that pHl-∆LNS localizes to synapses. pHl-∆LNS fluorescence reversibly quenches when imaged in a pH 5.5 buffer suggesting that pHl-∆LNS is properly inserted into the plasma membrane. (**e**) Average fluorescent intensity of pHl-∆LNS puncta was significantly reduced by imaging in pH 5.5 buffer and recued upon perfusion of original pH 7.3 imaging buffer. **p < 0.01, and ***p < 0.001 as determined by 1-way ANOVA and post hoc Tukey test. N = 34 pHl-∆LNS puncta. Scale bar = 1 μm.

To disrupt the function of all α- and β-Nrxns, we took two approaches. First, we designed Nrxn shRNA constructs that targeted each of the 6 primary Nrxn mRNA transcripts (Fig. 1a). shRNA constructs were designed to target mRNA sequences present in both α and β Nrxn transcripts so that a single shRNA should attenuate expression of both α and β isoforms for a particular Nrxn gene. The effectiveness of individual shRNA in reducing exogenous neurexin mRNA was first screened in a fluorescent assay in neurons (Supplemental Fig. S1). Effective shRNAs were then combined into two triple knockdown vectors (TKD1 and TKD2), each with unique shRNA sequences target towards Nrxn 1, 2, and 3 mRNA (Fig. 1a, right). Neurexin TKD1 and TKD2 constructs were further evaluated by co-transfecting mGFP fusion-tagged constructs of Nrxn 1β, 2β, and 3β along with Ctrl, TKD1 or TKD2 plasmids into HEK293 cells and performing a western blot with an anti-GFP antibody (Fig. 1b; for original Western blots see Supplemental Fig. S2).

In a second approach to disrupt the function of all Nrxns, we created a mutant Nrxn-1β construct in which the LNS domain essential for binding neuroligins and LRRTMs was deleted (Nrxn-1β∆LNS, Fig. 1c). To characterize the subcellular localization of Nrxn-1β∆LNS, we inserted the pH-sensitive fluorescent protein, superecliptic pHluorin (pHl) in place of the extracellular LNS domain to create pHl-Nrxn-1β∆LNS (pHl-∆LNS, Fig. 1c–e). pHl-∆LNS co-localized with synaptophysin-mCherry (Syph-mCh) puncta at putative presynaptic specializations (Fig. 1d). To test whether the pHl-∆LNS construct was properly inserted into the plasma membrane, we imaged pHl-∆LNS expressing axons before, during, and after perfusion with a pH 5.5 buffer. The average fluorescent intensity of pHl-∆LNS puncta was significantly reduced during perfusion of the pH 5.5 buffer and increased upon reperfusion with pH 7.3 buffer (Fig. 1e. **p < 0.01, and ***p < 0.001 as determined by 1-way ANOVA and post hoc Tukey test). Taken together, these findings suggest that the pHl-∆LNS construct is correctly inserted into the plasma membrane at presynaptic specializations.

**Figure 2:**
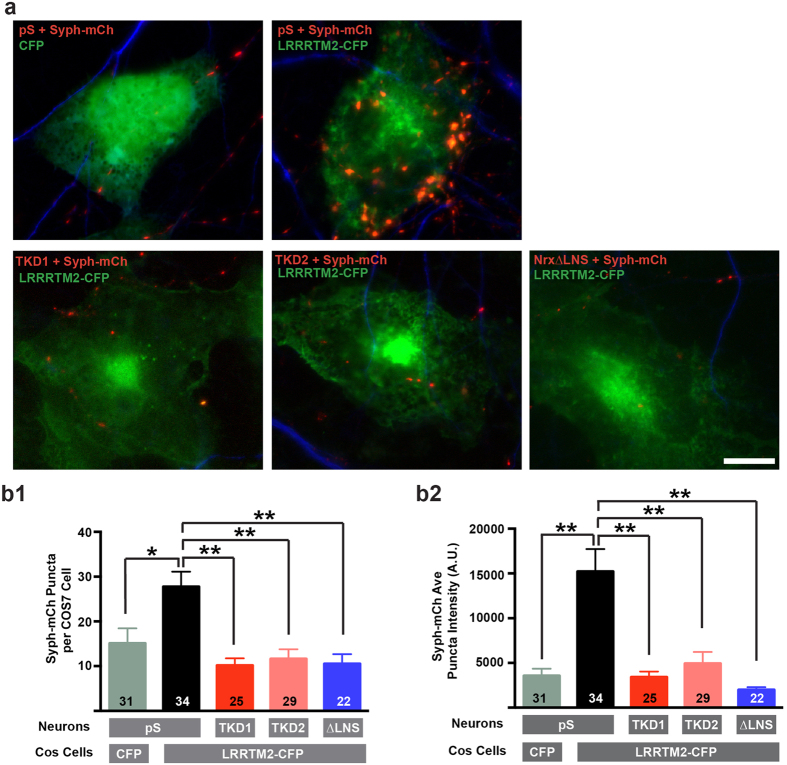
Perturbation of Nrxn function blocks the synaptogenic effect of LRRTM2 in co-culture synapse formation assay. (**a**) COS7 cells expressing CFP or LRRTM2-CFP cultured with hippocampal neurons expressing either an empty knockdown vector (pS), NrxnTKD1, NrxnTKD2, or Nrxn-1β∆LNS along with Synaptophysin-mCherry (Syph-mCh, red). CFP in COS7 cells is pseudo-colored green to allow for better detection of anti-MAP2 stained dendrites, shown in blue. (**b**) Quantification of Syph-mCh puncta density and intensity. LRRTM2-induced clustering of Syph-mCh puncta in NrxnTKD1, NrxnTKD2, or Nrxn-1β∆LNS expressing axons was significantly reduced compared to axons expressing an empty knockdown vector when quantified as average number of Syph-mCh puncta per COS7 cell (**b1**) or as average Syph-mCh cluster intensity per COS7 cell (**b2**; *p < 0.05, **p < 0.01, as determined by 1-way ANOVA and post hoc Tukey test). Numbers of COS7 cells analyzed are indicated in the graphs. Data are shown as mean+/− SEM. Scale bar indicates 10 μm.

To confirm that knockdown and Nrxn-1β∆LNS constructs perturb the function of endogenous neurexins in neurons, we co-cultured neurons transfected with knockdown or Nrxn-1β∆LNS constructs with fibroblasts expressing the neurexin ligand LRRTM2. Non-neuronally expressed LRRTM2 induces the recruitment of presynaptic specializations, or hemisynapses, in contacting axons in a neurexin-dependent manner^16^,^32^. Functional perturbation of endogenous neurexins should inhibit the formation of hemisynapses at contact sites with LRRTM2 expressing fibroblasts. We co-cultured COS7 cells with dissociated hippocampal neurons that were transfected with either an empty knockdown vector (pS), NrxnTKD1, NrxnTKD2 or Nrxn-1β∆LNS. Synaptophysin-mCherry (Syph-mCh) was included in the neuronal transfection as a fluorescent marker of synaptic vesicles to detect hemisynapses. After 48 hours, co-cultures were fixed and neuronal dendrites were immunostained with an antibody raised against MAP2 to exclude Syph-mCh puncta made onto dendrites from the analysis. LRRTM2-CFP expression in COS7 cells induced dramatic clustering of Syph-mCh puncta in control axons expressing an empty knockdown vector, in contrast to COS cells expressing a CFP control vector (Fig. 2a). Compared to control axons, expression of NrxnTKD1, NrxnTKD2 or Nrxn-1β∆LNS in neurons significantly reduced both the average number of Syph-mCh puncta per COS7 cell (Fig. 2b1, *p < 0.05, **p < 0.01, one-way ANOVA and post hoc Tukey test), as well as the average intensity of Syph-mCh puncta per COS7 cell (Fig. 2b2, p < 0.01, one-way ANOVA and post hoc Tukey test). These data suggest that shRNA constructs diminish endogenous Nrxn expression and that our Nrxn-1β∆LNS acts in a dominant negative fashion to perturb Nrxn function, presumably by inhibiting the binding of endogenous α- and β-Nrxns to presynaptic scaffolding proteins such as the CASK/Mint1/Veli complex^33^,^34^.

### Perturbation of Nrxn function reduces readily releasable pool size and attenuates neurotransmitter release probability

Electrophysiological studies of Nrxn gene knockouts have indicated isoform-specific roles of Nrxns in the modulation of neurotransmitter release^24^,^25^. In contrast, several studies that have disrupted the function of one or several postsynaptic Nrxn ligands have failed to find evidence for changes in presynaptic function^27^,^35^,^36^, questioning the notion of a transsynaptic modulation of neurotransmitter release through postsynaptic Nrxn ligands^37^. To test the role of Nrxns in the modulation of neurotransmitter release, we used a genetically encoded sensor, synaptophysin-pHluorin (SypHl), to quantify synaptic vesicle exocytosis at synapses from neurons with attenuated Nrxn function. In comparison to electrophysiological recordings, this optical method has the advantage of probing presynaptic function directly, in isolation from any effects of Nrxn perturbation on synaptic density and the recruitment of postsynaptic neurotransmitter receptors.

**Figure 3:**
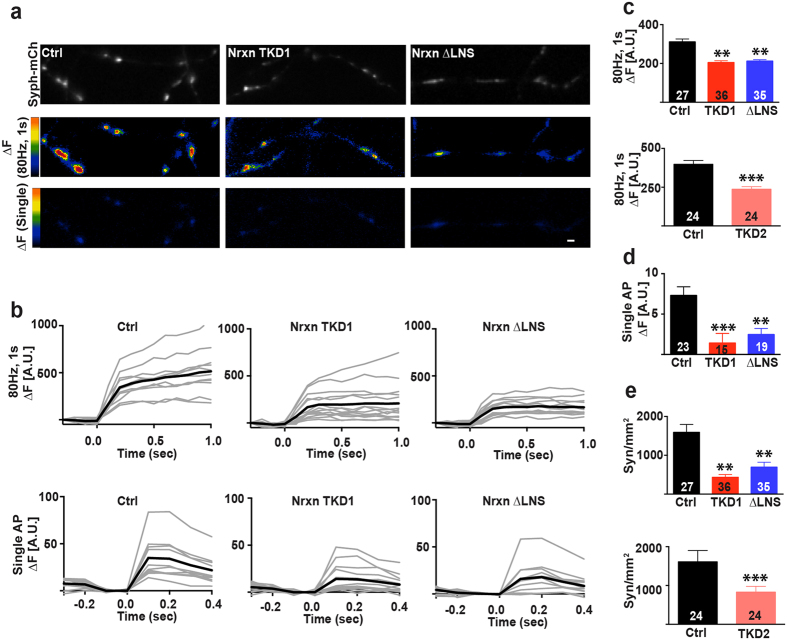
Nrxn perturbation attenuates neurotransmitter release. (**a**) Upper panel: synaptophysin-mCherry (Syph-mCh) expressing axons for control, NrxnTKD1 and Nrxn-1β∆LNS groups. Syph-mCh puncta were used to identify transfected axons. Middle Panel: SypHl fluorescence change (∆F) that occurs during high frequency stimulation, ∆F(80 Hz, 1 s), which exhausts the readily releasable pool of synaptic vesicles. Lower panel SypHl fluorescence change that occurs during single stimulation (∆F(Single). (**b**) SypHl fluorescent traces for micrographs presented above. Upper panel: SypHl fluorescence traces during high frequency stimulation (∆F(80 Hz, 1 s) for control, NrxnTKD1 and Nrxn-1β∆LNS groups. Lower panel: SypHl fluorescence traces during single stimulation (∆F(Single) for control, NrxnTKD1 and Nrxn-1β∆LNS groups. Gray traces show SypHl ∆F at individual synapses (high frequency stimulation averaged over 4 trials; single stimulation averaged over 180 trials). Black traces show the average synaptic SypHl response. (**c**) Average SypHl fluorescence changes per experiment in response to high frequency stimulation for NrxnTKD1/Nrxn-1β∆LNS experiments (upper graph) and for NrxnTKD2 experiments (lower graph). (**d**) Average SypHl fluorescence changes per experiment in response to single stimulation for NrxnTKD1/Nrxn-1β∆LNS experiments. (**e**) Density of high frequency SypHl fluorescent responses for NrxnTKD1/Nrxn-1β∆LNS experiments (upper graph) and for NrxnTKD2 experiments (lower graph). **p < 0.01 and ***p < 0.001 as determined by 1-way ANOVA and post hoc Tukey test. Data are shown as mean+/− SEM. Number of independent experiments are indicated in the respective graphs. Number of analyzed puncta = 1919, 697, and 1086 for Ctrl, NrxnTKD1, and Nrxn-1β∆LNS respectively in the upper panels of Fig. 3c and e. Number of analyzed puncta = 1727 and 887 for Ctrl and NrxnTKD2 respectively in the lower panels of Fig. 2c and e. Number of analyzed puncta = 1116, 297, and 502 for Ctrl, NrxnTKD1, and Nrxn-1β∆LNS respectively in Fig. 3d. Scale bar = 1 μm.

Cultures of dissociated hippocampal cells were transfected at 12–14 DIV with either Nrxn knockdown or Nrxn-1β∆LNS plasmids or a knockdown vector devoid of shRNA sequences, along with SypHl. Synaptophysin-mCherry (Syph-mCh) was included in the transfection mixes and was used to identify transfected axons (Fig. 3a, top panel). A high-frequency stimulus train (80 Hz for 1 s) was used to compare the size of the readily releasable pool (RRP) of synaptic vesicles at synapses from neurons expressing NrxnTKD1, Nrxn-1β∆LNS or empty knockdown vector (Fig. 3a, middle panel; Fig. 3b, upper graph). The SypHl fluorescence increase in response to high-frequency train stimulation, ∆F(80 Hz, 1 s), was reduced by 34% and 32% in NrxnTKD1 and Nrxn-1β∆LNS groups, respectively, compared to control cultures (Fig. 3c, upper graph), indicating a reduction of RRP size in both groups (p < 0.01, one-way ANOVA and post hoc Tukey test). To ensure that the effects of NrxnTKD1 shRNA on RRP size are not due to off-target effects of the shRNA, we performed a separate set of experiments using NrxnTKD2, a KD plasmid with 3 different shRNA sequences targeted towards Nrxn mRNA. Perturbation of Nrxn function with NrxnTKD2 resulted in a comparable reduction in peak SypHl fluorescence during high frequency stimulation (41% reduction, p < 0.001, Student’s t-test, independent samples, Fig. 3c. lower graph). At synapses that responded to high frequency stimulation, we measured the SypHl fluorescence increases in response to single action potentials, ∆F(1AP), a measure of the probability of neurotransmitter release at individual presynaptic specializations (Pr^38^,). Average SypHl fluorescence increases in response to isolated action potentials were strongly reduced in NrxnTKD1 and Nrxn-1β∆LNS groups (Fig. 3d). Compared to control synapses, ∆F(1AP) was reduced by 81% at NrxnTKD1 synapses and 66% at Nrxn-1β∆LNS synapses (**p < 0.01, ***p < 0.001, one-way ANOVA and post hoc Tukey test).

While the amplitude of SypHl fluorescence increases to high-frequency train stimulation allows an assessment of RRP size, the density of axonal varicosities with increases in SypHl fluorescence can serve as a measure of the density of synapses that release neurotransmitter. We therefore also analyzed the effect of Nrxn perturbation on the density of axonal sites with SypHl fluorescence increases in response to high frequency stimulation. Neurons expressing NrxnTKD1 and Nrxn-1β∆LNS groups showed a 73% and 56% reduction, respectively, in the density of axonal sites displaying a SypHl fluorescence increase in response to high-frequency stimulation as compared to control neurons expressing the knockdown vector lacking the shRNA (p < 0.01, one-way ANOVA and post hoc Tukey test, Fig. 3e, upper graph). Similarly, neurons expressing NrxnTKD2 displayed 49% reduction in the density of axonal sites with SypHl increases (p < 0.001, Student’s t-test for independent samples, Fig. 3e, lower graph). Taken together, these results suggest that disruption of Nrxn function causes a decrease in release probability that is partly due to a reduction in RRP size, as well as a reduction in the density of functional synapses.

### Nrxn disruption reduces active zone protein content and synaptic density

**Figure 4:**
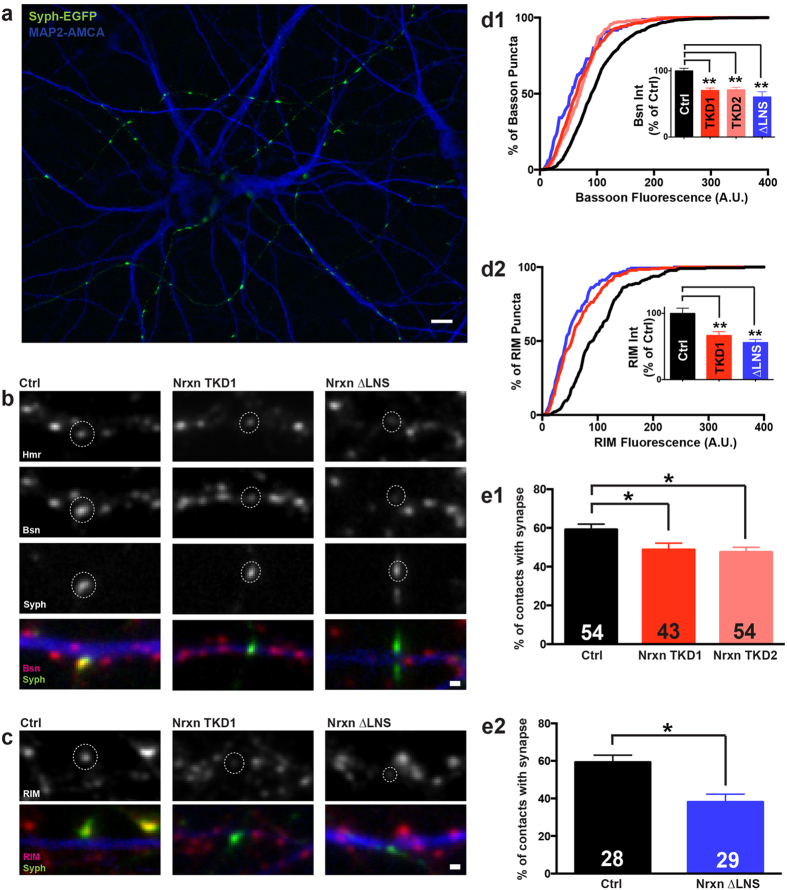
Nrxn disruption reduces active zone cytomatrix protein content and synaptic density. (**a**) Overview image of synaptic contacts between MAP2 positive dendrites (blue) and axons co-transfected with synaptophysin-EGFP (syph-EGFP, green) + experimental treatment (Ctrl plasmid, NrxnTKD1 or Nrxn-1β∆LNS). (**b–c**) Nrxn disruption decreases immunofluorescence of active zone cytomatrix proteins. (**b**) Upper 3 panels: representative images of Homer and Bassoon (Bsn) immunostains and syph-EGFP expressing axons for the 3 experimental groups. Circled puncta show Bsn and Homer clusters that correspond to the transfected axon. Bottom panel: Merged image of Bsn (red), Syph-EGFP (green) and MAP2 (blue) fluorescence. (**c**) Upper panel: RIM1/2 immunofluorescence for the 3 experimental groups. Bottom panel: Merged image of RIM (red), Syph-EGFP (green) and MAP2 (blue) fluorescence. (**d**) Cumulative histogram of immunofluorescence for Bsn (**d1**) and RIM1/2 (**d2**). Inset graphs show average puncta immunofluorescence, normalized to control. For Bsn immunofluorescence experiments, n = 89, 57, 38 and 30 postsynaptic neurons for Ctrl, TKD1, TKD2, and Nrxn-1β∆LNS groups respectively. For RIM immunofluorescence experiments, n = 25, 23, and 21 postsynaptic neurons for Ctrl, TKD1, and Nrxn-1β∆LNS groups respectively. **p < 0.01 as determined by 1-way ANOVA and post hoc Tukey test. (**e1–e2**) Nrxn disruption reduces synaptic density. Percentage of axodendritic contacts with both Bsn and Homer clusters per experiment for NrxnTKD1 (**e1**) and Nrxn-1β∆LNS overexpression (**e2**). The number of analyzed postsynaptic neurons is indicated in the respective graphs. *p < 0.05 as determined by 1-way ANOVA and post hoc Tukey test (For TKD experiments) and Student’s t-test (For Nrxn-1β∆LNS experiments). Data are shown as mean+/− SEM. Number of analyzed puncta = 894, 612, 181, and 85 for Ctrl, NrxnTKD1, NrxnTKD2 and Nrxn-1β∆LNS respectively in Fig. 4d1; 226, 190 and 138 for Ctrl, NrxnTKD1 and Nrxn-1β∆LNS respectively in Fig. 4d2; 759, 520 and 767 for Ctrl, NrxnTKD1 and NrxnTKD2 respectively in Fig. 4e1 and 636 and 708 for Ctrl and Nrxn-1β∆LNS respectively in Fig. 4e2. Scale bars = 10 μm (**a**), 1 μm (**b**–**c**).

As outlined above (Fig. 3), disruption of Nrxn function causes a reduction in the size of the readily releasable pool (RRP) of synaptic vesicles. It has previously been shown that RRP size correlates with the size of the active zone cytomatrix^38^,^39^, which presumably determines the number of docking sites available for synaptic vesicles. We therefore tested if perturbation of Nrxn function alters the recruitment of active zone cytomatrix proteins to presynaptic specializations. For this purpose, we transfected hippocampal cultures at 12–14 DIV with either empty KD vector, NrxnTKD1, NrxnTKD2 or Nrxn-1β∆LNS along with a plasmid encoding synaptophysin-EGFP (Syph-EGFP) to mark transfected axons and presynaptic specializations. Three days following the transfection, we performed immunocytochemistry to quantify the amount of the active zone cytomatrix proteins Bassoon (Bsn) and, in a separate set of experiments, Rab3-associated molecule 1/2 (RIM1/2). We also immunolabeled microtubule associated protein 2 (MAP2) to restrict our analysis to points of contact between transfected axons and MAP2-positive dendrites (Fig. 4a). We observed that NrxnTKD or overexpression of Nrxn-1β∆LNS significantly attenuated the intensity of Bsn and RIM puncta at axodendritic contacts (Fig. 4d). Average Bsn intensity was reduced by 29.8% with NrxnTKD, 28.5% with NrxnTKD2, and 39.4% with Nrxn-1β∆LNS (p < 0.01 as determined by 1-way ANOVA and post hoc Tukey test). Average RIM intensity per experiment was reduced by 33.3% with NrxnTKD and 44% with Nrxn-1β∆LNS (p < 0.01 as determined by 1-way ANOVA and post hoc Tukey test). Histograms for Bsn and RIM puncta intensity suggest that Nrxn perturbation results in a proportional reduction of Bsn and RIM at all synapses rather than a selective reduction at a subset of synapses. These results show that Nrxn is important for the recruitment of active zone cytomatrix proteins Bsn and RIM at axodendritic contacts and indicate that the reduction in neurotransmitter release in NrxnTKD1, NrxnTKD2 and Nrxn-1β∆LNS expressing neurons may partly be due to alterations in active zone cytomatrix size or composition.

Our experiments using SypHl to assess the density of functional presynaptic sites indicated that neurons with perturbed Nrxn function may have fewer synaptic contacts. To further investigate this possibility, we transfected neurons in hippocampal cultures with NrxnTKD1, NrxnTKD2 or Nrxn-1β∆LNS along with a plasmid encoding synaptophysin-EGFP to mark transfected axons. Since our transfection method results in sparse expression of the construct in 5–10% of all neurons, we were unable to employ the conventional methodology of measuring the density of dendritic spines in postsynaptic neurons. We therefore developed a novel approach to quantify synapse density on transfected cultures immunostained for Bsn, the postsynaptic protein Homer1, and MAP2. We initially identified points at which axons of transfected neurons traversed MAP2-positive dendrites. We then determined the fraction of contact points bearing co-localized Bsn and Homer1 puncta as a measure of the density of synaptic contacts made by transfected presynaptic neurons (Fig. 4a–c). Compared to control neurons, neurons expressing NrxnTKD1, NrxnTKD2 or Nrxn-1β∆LNS constructs displayed a significant reduction in the fraction of axodendritic contacts bearing synapses (Fig. 4e). In NrxnTKD experiments, the average percentage of synapse-bearing axodendritic contacts was 59.2+/−2.8% for Ctrl, 48.8+/−3.3% for NrxnTKD, and 47.6+/−2.5% for NrxnTKD2 (p < 0.05 as determined by 1-way ANOVA and post hoc Tukey test). In experiments assessing the effect of Nrxn-1β∆LNS on synaptic density, the average percentage of synapse-bearing axodendritic contacts per experiment was 59.4+/−3.7% for Ctrl and 38.2+/−4.1% for Nrxn-1β∆LNS (p < 0.05 for as determined by Student’s t-test). Together, these experiments demonstrate that functional perturbation of all Nrxns leads to a reduction in the density of glutamatergic synapses.

### Nrxn disruption reduces the stability of synaptic contacts

**Figure 5:**
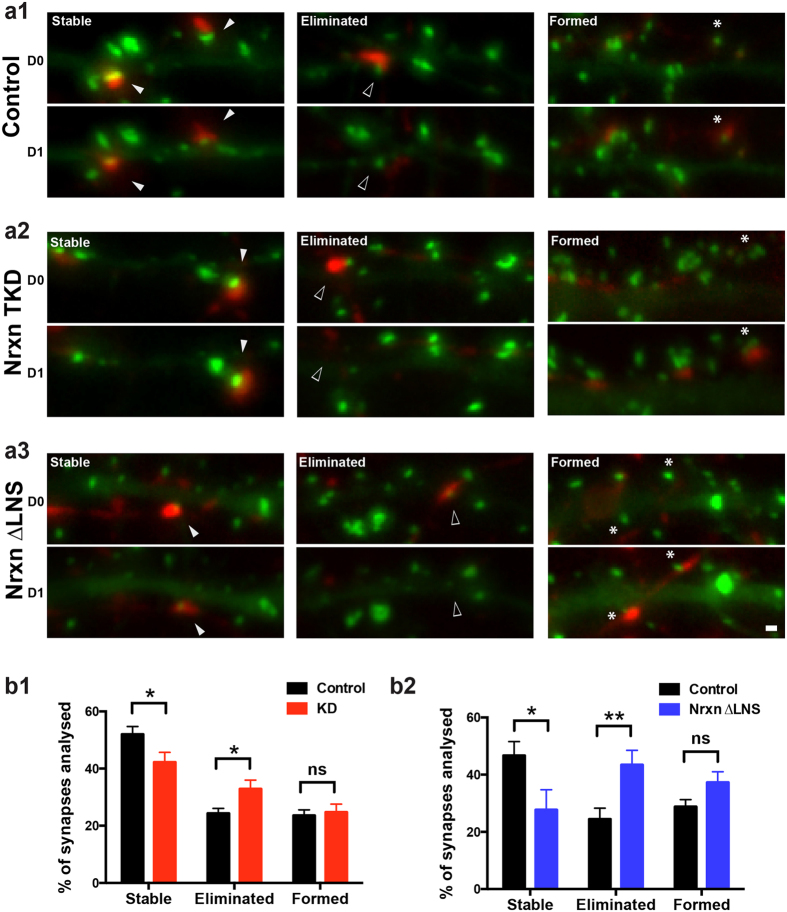
Nrxn disruption reduces the stability of synaptic contacts. (**a1–a3**) Example images of contacts between syph-mCherry expressing axons and PSD95-EGFP expressing dendrites imaged on Day 0 and 24 hrs later (Day1). Examples of stable (filled arrowheads), eliminated (open arrowheads), and formed synapses (asterisks) are shown for control, NrxnTKD1, and Nrxn-1β∆LNS groups. (**b1–b2**) Average percentage of stable, eliminated and formed synapses, grouped by postsynaptic cell for NrxnTKD1 (**b1**) and Nrxn-1β∆LNS (**b2**) experiments. *p < 0.05, **p < 0.01 as determined by Student’s t-test. In NrxnTKD1 experiments n = 41 (Ctrl) and 39 (NrxnTKD1) postsynaptic neurons. In Nrxn-1β∆LNS experiments, n = 14 (Ctrl) and 11 (Nrxn-1β∆LNS) postsynaptic neurons. Data are shown as mean + /−SEM. Number of analyzed synapses = 566 and 614 for Ctrl and NrxnTKD1 respectively in Fig. 5b,1 and 345 and 185 for Ctrl and Nrxn-1β∆LNS respectively in Fig. 5b2. Scale bar = 1 μm.

The reduced density of glutamatergic synapses we observed at contacts between Nrxn-perturbed axons and dendrites may be either due to a reduced rate of synapse formation or, alternatively, an increased rate of synapse elimination. To discern between these two possibilities, we performed live time-lapse imaging of fluorescently labeled hippocampal neurons. We first co-transfected empty knockdown, NrxnTKD1or Nrxn-1β∆LNS plasmids along with Syph-mCh to label presynaptic specializations. We then subsequently transfected PSD95-EGFP to label postsynaptic densities in a separate population of neurons. Co-localizations of Syph-mCh and PSD95-EGFP were imaged over a period of 24 hours and the percentage of stable, eliminated, and newly formed synapses was recorded in NrxnTKD1 and Nrxn-1β∆LNS groups and compared with control cultures. In all groups, we found examples of co-localized Syph-mCh and PSD95-EGFP that were stable, eliminated or newly formed during the observation period (Fig. 5a). Quantification of the percentage of stable, eliminated, and newly formed synaptic contacts showed that both NrxnTKD1 and Nrxn-1β∆LNS overexpression resulted in synaptic contacts that were significantly less stable compared to control cultures (Fig. 5b). In experiments employing NrxnTKD1 to perturb Nrxn function, the percentage of stable synapses per postsynaptic cell over 24 hours was 52.0+/−2.7% in Ctrl and 42.3+/−3.4% in NrxnTKD1 expressing axons (p < 0.05, Student’s t-test). In separate experiments using Nrxn-1β∆LNS, the percentage of stable synapses per postsynaptic cell was 46.7+/−4.9% in ctrl and 27.7+/−7.0% in Nrxn-1β∆LNS expressing neurons (p < 0.05, Student’s t-test). Interestingly, in both NrxnKD1 and Nrxn-1β∆LNS groups, rates of synapse formation were not significantly different from those in control cultures. In summary, these experiments provide evidence for the notion that Nrxns have an important role in the stabilization of synapses but are not essential for the formation of initial synaptic contacts.

## Discussion

In this study, we show that the Nrxn family of presynaptic cell adhesion molecules play an important role in the functional and structural integrity of synaptic contacts. Disruption of Nrxn function in hippocampal neurons, either by shRNA-mediated knockdown of all Nrxn isoforms or by overexpression of a mutant Nrxn unable to bind postsynaptic ligands, led to a reduction in neurotransmitter release probability at affected synapses. The perturbation of Nrxn function also caused a reduction of the density of glutamatergic synaptic connections. Importantly, using time-lapse imaging of synaptically connected neurons, we show that disruption of Nrxn function increases the rate of synapse elimination but does not affect the rate of synaptogenesis. Our data therefore suggest a prominent function of these presynaptic cell adhesion proteins in the stabilization and functional maturation of glutamatergic synapses.

By using an optical method to directly measure synaptic vesicle exocytosis in cultured hippocampal neurons, we demonstrate that release probability is strongly reduced at synapses with attenuated Nrxn function. This finding is consistent with electrophysiological evidence from previous studies indicating that a reduction in neurotransmitter release probability at glutamatergic synapses in mice deficient in either all α- or all β-Nrxns^24^,^25^. Our results further suggest that the attenuation of neurotransmitter release probability in Nrxn-depleted neurons is in part due to a strong reduction in the size of the readily releasable pool of synaptic vesicles. The reduction in RRP size we observed with pan-Nrxn knockdown was more pronounced than the decrease in α-Nrxn specific knockout neurons^24^ and in contrast to unchanged pools of readily releasable synaptic vesicles in β-Nrxn deficient neurons^25^. To enter the RRP, synaptic vesicles have to dock to the plasma membrane and undergo SNARE complex formation, or priming. These processes crucially depend on the availability of active zone cytomatrix components that provide synaptic vesicle docking sites and facilitate priming. Interestingly, we show here that two components of active zone cytomatrices, Bsn and RIM, are reduced at synapses made by neurons with perturbed Nrxn function. This finding suggests that the decrease of RRP size observed with disruption of Nrxn function may be secondary to diminished recruitment of active zone cytomatrix components, which then causes a reduction in synaptic vesicle docking and priming.

With this study, we further sought to clarify the role of Nrxns in synapse formation. Co-culture studies have shown that Nrxns and Nrxn ligands are able to recruit components of pre- and postsynaptic specializations to synapses, suggesting a synaptogenic function of these proteins. However, isoform-specific knockouts of either all α- or all β-Nrxn did not lead to reductions in synaptic density, suggesting that these cell adhesion proteins are potentially dispensable for synaptogenesis^24^,^25^. To examine the role of Nrxns in synaptic development, we interfered with the function of all Nrxn isoforms and assessed the effect of this manipulation on synapse density as well as on synaptic turnover. Immunocytochemical analysis of pre- and postsynaptic protein content showed that pan-Nrxn KD or overexpression of Nrxn-1β∆LNS results in a significant reduction in the density of glutamatergic synapses. This result is consistent with previous studies demonstrating that overexpression of neuroligins increases the density of glutamatergic synapses, while the attenuation of neuroligin and LRRTM2 expression decreases their density^28^,^40^. To address whether this reduction is the result of a decreased rate of synapse formation or due to increased synapse elimination, we carried out time-lapse imaging of synaptically connected hippocampal neurons. Surprisingly, we found that Nrxn perturbation did not affect the rate of synapse formation but instead increased the elimination of preexisting synaptic contacts.

**Figure 6:**
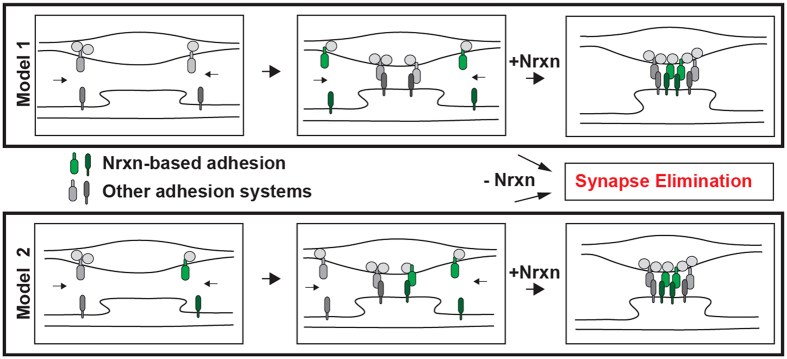
Model 1 (upper panel) The initial formation of synaptic contacts is mediated by synaptic cell adhesion proteins other than Nrxns. Nrxns and/or postsynaptic Nrxn ligands may be incorporated into synapses only during their maturation, and may prevent elimination of nascent synapses at this stage. Model 2 (lower panel) Nrxns are co-expressed with other cell adhesion proteins and function redundantly before the formation of synaptic contacts to induce synapse formation. Elimination of one class of adhesion molecules has little effect on the rate of synapse formation, because other cell adhesion proteins are able to fully compensate. However, the attenuation of transsynaptic cell adhesion during synaptic maturation may place synapses deficient in any individual cell adhesion protein at a disadvantage and favor their elimination.

These results indicate that Nrxns function in the stabilization and maturation of already formed synaptic contacts and are consistent with two alternative models (Fig. 6). In the first model, the initial formation of synaptic contacts between cortical neurons is mediated by synaptic cell adhesion proteins other than Nrxns. Nrxns and/or postsynaptic Nrxn ligands may be incorporated into synapses only during their maturation, and may prevent elimination of nascent synapses at this stage. In an alternative scenario, Nrxns are co-expressed with other families of cell adhesion proteins, such as receptor protein tyrosine phosphatases, before the formation of synaptic contacts and induce synapse formation in a highly redundant manner. In this model, which is also consistent with the findings of studies showing formation of presynaptic specializations in axons contacting non-neuronal cells expressing Nrxn ligands^16^,^18^,^19^,^23^; elimination of one class of cell adhesion proteins has little effect on the rate of synapse formation, because other cell adhesion proteins are able to fully compensate. However, the attenuation of transsynaptic cell adhesion during synaptic maturation may place synapses deficient in any individual cell adhesion protein at a disadvantage and favor their elimination. To conclusively distinguish between these alternative models, better insight into the role of other families of synaptic cell adhesion proteins in synapse stabilization is required.

## Methods

### Generation of shRNA Nrxn knockdown constructs

Short-hairpin RNA sequences were designed using software from the Whitehead Institute (http://sirna.wi.mit.edu). Selected shRNA targeted mRNA sequences are common to both α- and β-Nrxn isoforms. The KD efficiency of each shRNA construct was quantified using a fluorescence assay described in the supplementary methods. shRNA sequences were combined into 2 Nrxn triple knockdown vectors (NrxnTKD1 and NrxnTKD2), each with unique shRNA sequences. The NrxnTKD1 construct encoded shRNAs for targets 5′-AATAGCCAAGCAACCATAATA-3′, 5′-GTGTCCAAGTGATGATGAG-3′, and 5′-CAGTCTCGGGAACAACACATA-3′ for knockdown of Nrxn1, Nrxn2, and Nrxn3, respectively. The NrxnTKD2 construct contained shRNA sequences for the targets 5′-GGACAGATGACATCGCCATTG-3′, 5′-GAACACAGATGACCTTCTG-3′, and 5′-AAGTCTCGGAAACTAGTAGAA-3′ for knockdown of Nrxn1, Nrxn2, and Nrxn3, respectively. shRNA sequences were driven by either U6 or H1 promoters (see Fig. 1a).

### cDNA constructs

To create the mGFP-Nrxn fusion constructs used for Western blotting, cDNAs for Nrxn-1β (M96375), Nrxn-2β (M96377, with amino acids 203–232 and 368–561 spliced out), and Nrxn-3β (XM_008764732, with amino acids 202–231 spliced out and with nucleotides 968–979 from start ATG absent) were cloned in frame at the 3′ end of the mGFP cDNA in a vector allowing for the expression of the construct from a CMV promoter. Nrxn-1β∆LNS was created by deleting the LNS domain of Nrxn-1β using site-directed mutagenesis with the primers 5′-GGAATACGTCGTCCCAGCGT-GTC-3′ and 5′-GGAAGGCTGGTCGGTGAAGTGC-3′. This PCR removed amino acids 87–285 (UniProtKB - Q63373) and inserted a BspEI site in its place. pHluorin-Nrxn-1β∆LNS was created by inserting an AgeI, BspEI excised pHluorin cassette into the Nrxn-1β∆LNS BspEI site. Synaptophysin-EGFP and Synaptophysin-mCherry constructs were generated by fusing the ORF of synaptophysin (obtained via RT-PCR) in-frame to the 5′ end of the EGFP/mCherry cDNA contained in a pEGFP-C1-based expression vector. Synaptophysin-pHluorin (SypHl) has been previously described^38^. LRRTM2-CFP was a gift from Dr. Ann Marie Craig. CMV promoters were used to drive the expression of all exogenous sensors and constructs.

### Dissociated Hippocampal Cultures and Neuronal Transfection

Dissociated neuronal cultures were prepared from E18 embryonal Sprague Dawley rat hippocampi. All experiments on animals were approved by the Dalhousie University Committee on Laboratory Animals (UCLA Protocol #15–113) and performed in accordance with the approved guidelines. Cultures were grown in Neurobasal supplemented with B27 (Thermofisher, Waltham MA). For all experiments, cultures of hippocampal neurons were transfected 10–14 days after plating using a calcium-phosphate precipitation protocol. Imaging experiments were conducted 3–4 days after transfection. For additional detail on culture and transfection experimental procedures please see ref. 38.

### Immunocytochemistry and Western Blotting

Dissociated neuronal cultures were fixed with 4% paraformaldehyde at room temperature for 3 min followed by methanol at 4 °C for 10 minutes. Coverslips were washed with PBS, transferred onto parafilm wax, and blocked with 1% bovine serum albumin and 0.3% gelatin in PBS for 1 hr. Primary antibodies were diluted in blocking solution and applied for 24 hrs at room temperature. Primary antibodies include: Bassoon (Mouse monoclonal Ab, Enzo, SAP7F407, 1:400), Homer1 (Rabbit polyclonal Ab, Synaptic Systems, Lot # 160002, 1:3000), RIM1/2 (Rabbit polyclonal Ab, Synaptic Systems, Lot # 140203, 1:1000) and MAP2 (Guinea Pig polyclonal Ab, Synaptic Systems, Lot # 188004, 1:400). Coverslips were then washed with PBS and blocked for 30 minutes. Fluorescently labeled secondary antibodies were diluted in blocking solution and applied for 1 hr at room temperature. Secondary antibodies include: Alexa Fluor 660 (Goat anti-mouse, 1:1200), DyLight 549 (Donkey anti-rabbit 1:400), and AMCA (Donkey anti-guinea pig, Jackson Labs, 1:400). Coverslips were washed with PBS and mounted onto slides using Aqua-Mount (Thermo Scientific).

For western blotting, HEK 293 cells were transfected using polyethylenimine with1 μg mGFP-Nrxn-1β, mGFP-Nrxn-2β, or mGFP-Nrxn-3β constructs and 10 μg of either an empty knockdown plasmid, NrxnTKD1, or NrxnTKD2 per 35 mm dish. 10 μg of lysed samples per lane were loaded on an 8% SDS-PAGE gel and then transferred to nitrocellulose membranes. Samples were blocked for 1 hour at room temperature with blocking solution (5% non-fat dry milk and 0.1% Tween-20 in TBS) and then incubated with primary antibodies overnight at 4 °C. Membranes were washed three times with 0.1% Tween-20 in TBS, blocked for 30 minutes and then incubated with HRP conjugated secondary antibodies for 1 hour at room temperature. HRP signals were detected using a chemiluminescence solution (Bio-Rad, Cat. # 170–5060). Primary antibodies used were anti-GFP (Rabbit polyclonal, Synaptic Systems, Cat # 132002, dilution of 1:5000) and anti-Tubulin (Mouse monoclonal, Sigma, 6-11B-1 at a dilution of 1:10,000). Secondary antibodies included Donkey Anti-Rabbit HRP (Jackson, 711-035-152, 1:10000) and Donkey Anti-Mouse HRP (Jackson, 715-035-150, 1:10000).

### Characterization of pHl-Nrxn-1β∆LNS

Cultures of dissociated hippocampal neurons were transfected at 10–14 DIV with the pHl-Nrxn-1β∆LNS (pHl-∆LNS) construct. After 3 days, cells were imaged on a Zeiss Observer 2.1 inverted microscope using a 63x objective, Photometrics Coolsnap HQ2 camera and SlideBook 6 imaging software. Image stacks of pHl-∆LNS transfected axons were first acquired in pH 7.3 HBS buffer (described above). Neurons were then perfused with a pH 5.5 buffer (Composition in mM: 124 NaCl, 3 KCl, 10 MES, 5 D-glucose) for 5 minutes at which point a second image stack was acquired. Neurons were then re-perfused with the original pH 7.3 HBS buffer and a final image stack was acquired. Image stacks were converted to projected images according to maximum fluorescence and pHl-∆LNS fluorescent puncta were segmented and measured using IPLab software.

### Neuronal-COS7 Co-culture Assay

At 10 DIV, dissociated hippocampal neurons were transfected with 60 μg of either an empty KD vector, NrxnTKD1, NrxnTKD2, or Nrxn-1β∆LNS per 60 mm dish along with 25 μg Synaptophysin-mCherry (Syph-mCh) to label presynaptic specializations and serve as a measure for presynaptic clustering. Following transfection, coverslips were transferred to 12-well plates. The next day, COS7 cells were transfected using calcium phosphate precipitation with 30 μg of CFP or LRRTM2-CFP per 35 mm well. After 24 hrs of expression, COS7 cells were trypsinized, pelleted, and re-suspended in conditioned neuronal media. COS7 cells were then seeded onto neurons at a density of approximately 10,000 cells per well of a 12-well plate. Co-cultures were maintained for 48 hours and then fixed and immunostained with anti-MAP2 to label neuronal dendrites. In regions were Syph-mCh expressing axons contacted transfected COS7 cells, image stacks of 2.8 μm were acquired with a Zeiss Observer 2.1 inverted microscope using a 63x objective, Photometrics Coolsnap HQ2 camera and SlideBook 6 imaging software. Maximum projection images were created and exported for analysis. Images were analyzed for the number and intensity of Syph-mCh puncta per transfected COS7 cell using IPLab software. Contacts between Syph-mCh expressing axons and MAP2 positive dendrites were excluded from analysis. Images were acquired from 2 independent co-cultures.

### Synaptophysin-pHluorin experiments

Cultures of hippocampal neurons were transfected with SypHl to quantify synaptic vesicle exocytosis as described^38^. 80 μg of either a control plasmid, NrxnTKD1, NrxnTKD2 or Nrxn-1β∆LNS were transfected along with 80 μg of SypHl. The presynaptic marker Syph-mCh was also included in the transfection to identify transfected axons. Fluorescence microscopy was carried out on a Nikon TE2000 epifuorescence microscope equipped with a 60x (N.A. 1.40) objective, Smart shutter (Sutter Instruments) and Lumencor solid-state illumination. Images were acquired at 10 Hz with a Hamamatsu ORCA CCD camera and IPLab software. Experiments were performed at 36 ± 2 °C in HBS solution containing (in mM) 110 NaCl, 5.3 KCl, 2 CaCl2, 1 MgCl2, 20 4-(2-hydroxyethyl)-1-piperazineethanesulfonic acid (HEPES), and 25 D-glucose adjusted to pH 7.30, supplemented with 10 μM 6,7-dinitroquinoxaline-2,3-dione (DNQX) and 50 μM (2R)-amino-5-phosphonovaleric acid (APV) to prevent recurrent excitation. Axons were selected based on Syph-mCh fluorescence, and SypHl fluorescence changes were measured in response to field stimulation employing 1 ms square current pulses yielding electrical fields of approximately 10 V/cm through platinum electrodes placed 0.5 cm apart. Image acquisition and extracellular stimulation were synchronized using a Master-8 stimulator (AMPI). Stimulus trains of 80 stimuli at 80 Hz were given to measure the readily releasable pool of synaptic vesicles. In this study, we chose a higher stimulus number and stimulation frequency to deplete the RRP than in earlier studies by us and others^38^,^41^,^42^ to maximize mobilization of readily releasable vesicles while minimizing contributions of RRP refilling to the signal. Fluorescence increases in response to isolated stimuli (100 trials at 0.2 Hz) provided a relative measure of release probability (Pr) at individual presynaptic specializations. Images were acquired from at least two individual hippocampal cultures. Subsequent image analysis was performed using IPLab software. Image stacks were background-subtracted and aligned. For SypHl RRP and Pr, experiments, synaptic regions for measurement were identified according to an RRP threshold. Synaptic regions of measurement had a size between 0.32 and 0.64 μm^2^. Multi-trial fluorescent responses at each synapse were averaged and an average synaptic response for each experiment was calculated. Data were expressed as change in fluorescence (∆F). The statistical significance ∆F values for Ctrl, NrxnTKD1, and Nrxn-1β∆LNS groups was tested with a one-way ANOVA and post hoc Tukey tests.

### Assessment of active zone size and synaptic density

To assess the effect of Nrxn disruption on active zone size and synaptic density, we performed immunocytochemistry on cultures of transfected hippocampal neurons. Neurons were transfected with three cDNA plasmids: 1) either TKD1, TKD2, Nrxn-1β∆LNS, or control plasmid, 2) synaptophysin-EGFP (syph-EGFP), a fusion protein that labels clusters of synaptic vesicles and 3) cytosolic EGFP, as an axonal fill stain. Three days after transfection, neurons were immunostained for Bsn, RIM1/2 and MAP2 to assess active zone size and for Bsn, Homer1 and MAP2 to quantify synaptic density. Experimenters were blinded to the experimental conditions during image acquisition and analysis. Fluorescence images were taken on a Nikon TE2000 epifluorescence microscope equipped with a 60 X (N.A. 1.40) objective and Hamamatsu camera (Model C4742-80-12AG). To quantify the effect of Nrxn disruption on the size of active zone cytomatrices, we measured the fluorescence intensity of Bsn and RIM1/2 puncta (segment size = 0.5 μm^2^) that co-localized with syph-EGFP + EGFP expressing axons and MAP2 positive dendrites. Group averages of Bsn and RIM1/2 fluorescence were compared using either a one-way analysis of variance (ANOVA, for Ctrl, TKD1, TKD2) or a two-tailed Student’s t-test for independent samples (for Ctrl and Nrxn-1β∆LNS).

To quantify the effect of Nrxn disruption on synaptic density, we identified points at which axons of transfected neurons traversed MAP2-positive dendrites. We then determined the fraction of contact points bearing co-localized Bsn and Homer1 puncta as a measure of the density of synaptic contacts made by the transfected presynaptic neuron. An intensity threshold was set for both Homer1 and Bsn and kept constant for each experiment for a given transfected neuronal culture. Axodendritic contacts with both Homer1 and Bsn puncta intensities above threshold were scored as synaptic and contacts that failed these criteria were scored non-synaptic. Averages of the percentage of synaptic axodendritic contacts in each transfected culture were compared between groups using a one-way analysis of variance (ANOVA). Fasciculated neurites were excluded from analysis. Images were acquired from at least 2 separate hippocampal cultures.

### Quantification of synapse formation and elimination

To assess the effect of Nrxn perturbation on the stability of synaptic connections, we expressed fluorescently labeled pre- and postsynaptic proteins in hippocampal neurons and quantified the number of stable, newly formed and eliminated synapses over 24 hours. The presynaptic marker, synaptophysin-mCherry (Syph-mCh), was co-transfected with either a control plasmid, NrxnTKD1, or Nrxn-1β∆LNS at 13 day *in vivo* (DIV). In a second transfection at 15 DIV, PSD95-EGFP was introduced to label the postsynaptic densities in a separate population of neurons. On the following day, referred to in figures as day 0 (D0), coverslips were transferred to an imaging chamber, perfused with HBS solution at 36 ± 2 °C, and imaged with a Zeiss Observer 2.1 inverted microscope using a 63x objective, Photometrics Coolsnap HQ2 camera and SlideBook 6 imaging software. Stacks comprising 12 images over a distance of 3.85 μm were acquired for experiments that showed Syph-mCh positive axons in contact with PSD95-EGFP positive dendrites. The XY coordinates of each experiment were recorded and coverslips were returned to the incubator. The following day, a second image stack was acquired for each experiment (D1). Image stacks were converted to a maximum intensity projected image and exported for analysis. D0 and D1 images were first analyzed individually to detect synaptic contacts between Syph-mCh positive axons and PSD95-EGFP positive dendrites in control, NrxnTKD1, and Nrxn-1β∆LNS groups. The co-localization of a Syph-mCh punctum and a PSD95-EGFP punctum was scored a synapse if the center-to-center distance between both puncta was less than 0.8 μm. Next, the corresponding D1 image was aligned to the D0 image and the number of stable, newly formed, and eliminated synapse was quantified. Analysis was performed using IPLab software. Experiments with NrxnTKD1 construct and the Nrxn-1β∆LNS construct were performed independently. Using a Student’s t-test for independent samples, the percentage of stable, newly formed, and eliminated synapses were compared between NrxnTKD1, Nrxn-1β∆LNS and the respective control groups. Images were acquired from 6 separate hippocampal cultures for NrxnTKD1 experiments and 2 separate hippocampal cultures for Nrxn-1β∆LNS experiments.

## Additional Information

**How to cite this article:** Quinn, D. P. *et al*. Pan-neurexin perturbation results in compromised synapse stability and a reduction in readily releasable synaptic vesicle pool size. *Sci. Rep.* **7**, 42920; doi: 10.1038/srep42920 (2017).

**Publisher's note:** Springer Nature remains neutral with regard to jurisdictional claims in published maps and institutional affiliations.

## Supplementary Material

Supplementary Methods and Figures
