# Supplementary material for: Pan-neurexin perturbation results in compromised synapse stability and a reduction in readily releasable synaptic vesicle pool size
Source: Sci Rep. 2017 Feb 21;7:42920. doi: 10.1038/srep42920 (PMC5318902; doi:10.1038/srep42920)
Supplement: Supplementary Methods and Figures [file srep42920-s1.pdf]

Supplemental Information

Pan-neurexin perturbation results in compromised synapse stability and a reduction in readily releasable synaptic vesicle pool size

Dylan P. Quinn[1,2], Annette Kolar[1,2], Michael Wigerius[4], Rachel N. Gomm-Kolisko[3], Hanine Atwi[3], James P. Fawcett[4], and Stefan R. Krueger[1,2]

Supplementary Methods

cDNA target constructs for neuronal shRNA screen

cDNA segments of Nrxn 1, 2, and 3 were used as shRNA target sequences in a fluorescence-based screen for shRNA knockdown efficiency. The full-length cDNA for rat Nrxn-1β was obtained from Addgene (Plasmid 20173). Large cDNA fragments for rat Nrxn2 and Nrxn3 mRNA were obtained via RT-PCR. For Nrxn2, PCR primers 5'-ACCACTTCCACAGCAAGCAC-3' (forward) and 5'-GCGTAGAGAAGGATAAGGATGC-3' (reverse) were used to isolate a 967 bp fragment. For Nrxn3 primers 5'-GCGTTGACCATGCACCTGAG-3' (forward) and 5'-GTAAACATCACACCACCAGTCGTATGC-3' (reverse) were used to isolate a 1742 bp fragment. Nrxn cDNA fragments were ligated into an EGFP expression vector 3' to the EGFP stop codon, but 5' to the polyadenylation signal that defines the end of the mRNA, resulting in translation of only EGFP encoding parts of an mRNA also containing the neurexin target sequences. Target vectors also encoded TAG-BFP, which was expressed from a separate Cytomegalovirus (CMV) promoter to allow for normalization of cDNA expression. Target constructs for either Nrxn1, 2, or 3 were co-transfected into 10-13 DIV hippocampal neurons along with a shRNA KD vector or control vector. shRNA KD constructs that effectively targeted and degraded the EGFP-Nrxn transcript were identified by quantifying the intensity of somatic EGFP/BFP fluorescence intensity in KD neurons compared to control neurons which expressed an empty KD vector.

Supplementary Fig. 1

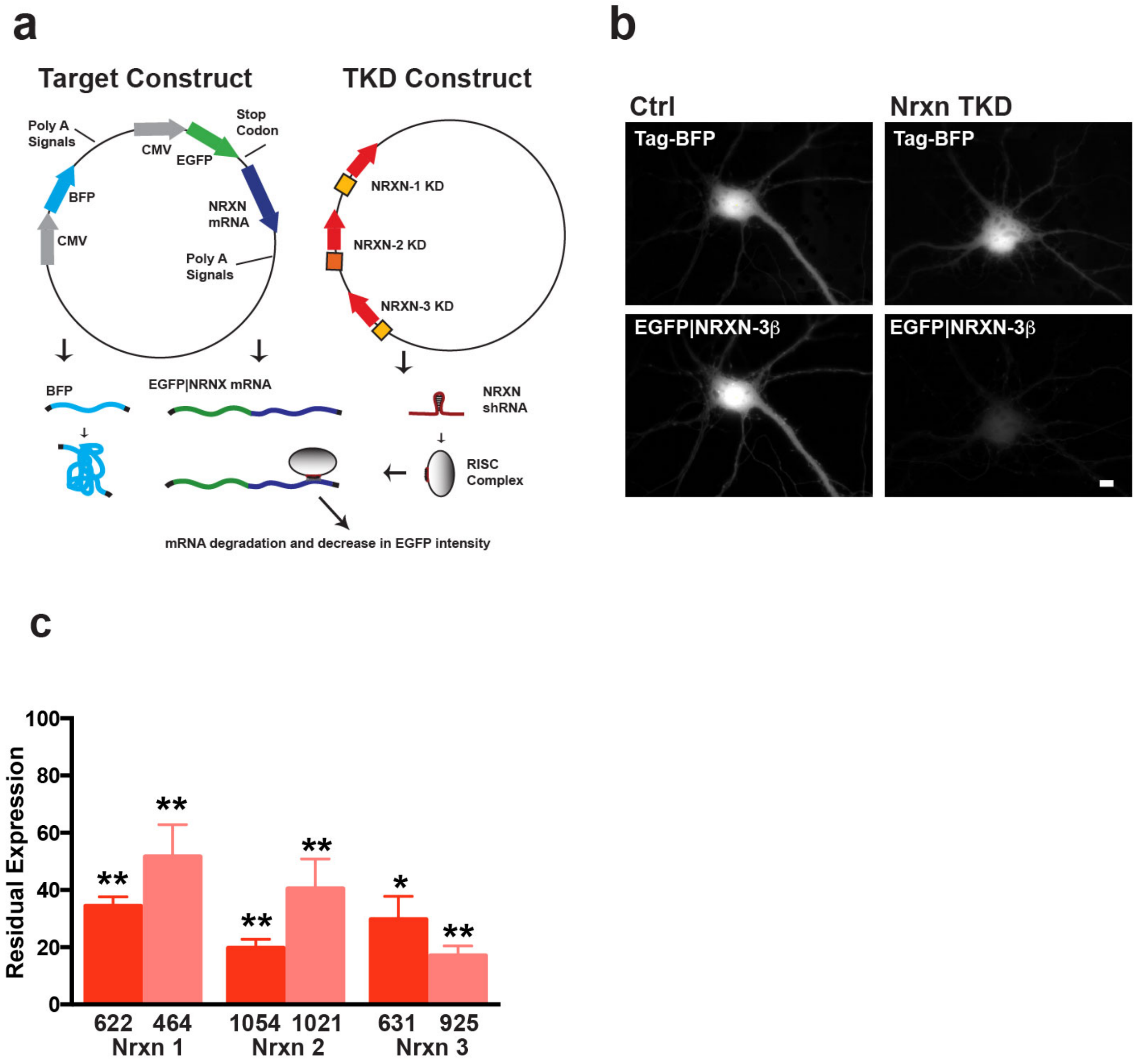

Fig S1. Neuronal shRNA knockdown screen. (A) To test the KD efficiency of candidate shRNA sequences we created target constructs for Nrxn1β, Nrxn2β, and Nrxn3β. Target constructs were generated from a vector that allows for expression of EGFP from a CMV promoter. Nrxn target sequences were ligated 3’ to the EGFP stop codon, but 5’ to the polyadenylation signal that defines the end of the mRNA. EGFP and Nrxn sequences are transcribed onto a single mRNA, which is targeted by candidate shRNA constructs. Unsuccessful targeting of shRNA to the Nrxn portion of the target mRNA results in translation of cytosolic EGFP as normal. Successful targeting of shRNA to the Nrxn portion of the target mRNA results in the degradation of the EGFP-Nrxn transcript and a reduction in cytosolic EGFP fluorescence. Knockdown efficiency was assessed in cultured hippocampal neurons by co-expressing EGFP|Nrxn1, 2, or 3 target constructs along with candidate shRNA sequences for each Nrxn gene. The Nrxn-EGFP target constructs also encoded blue fluorescent protein (Tag-BFP), driven by a separate promoter, to allow for normalization of transfection efficiency. (B) Neurons from a test of Nrxn-3 KDs. Knockdown efficiency was quantified by comparing somatic EGFP/BFP fluorescence intensities in Nrxn knockdown cells to that of controls cells that were co-transfected with an empty knockdown vector. shRNAs that were effective at reducing Nrxn expression where combined

into 2 triple knockdown vectors (TKD1 and TKD2), each with unique shRNA sequences target towards Nrxn 1, 2, and 3 mRNA. NrxnTKD1 and NrxnTKD2 were then retested in the fluorescent assay described above. (C) For NrxnTKD1, the residual fluorescent intensity of EGFP|Nrxn constructs was 34.4, 19.7, and 29.8% for EGFP|Nrxn 1, 2, and 3 respectively. In neurons expressing NrxnTKD2, the residual fluorescent intensity of EGFP|Nrxn constructs was 52.7, 40.5, and 17.1% for EGFP|Nrxn 1, 2, and 3 respectively. For Nrxn-1 experiments n's = 29, 15, and 12 cells for ctrl, sh-622, and sh-464 groups respectively. For Nrxn-2 experiments n's = 17, 18, and 14 cells for ctrl, sh-1054, and sh-1021 groups respectively. For Nrxn-3 experiments n's = 12, 10, and 10 cells for ctrl, sh-631, and sh-925 groups respectively. $^{*}p < 0.05$, $^{**}p < 0.01$ as determined by 1-way ANOVA and post hoc Tukey test. Data are shown as mean +/- SEM. Scale bar = 10 μm.

Supplementary Fig. 2

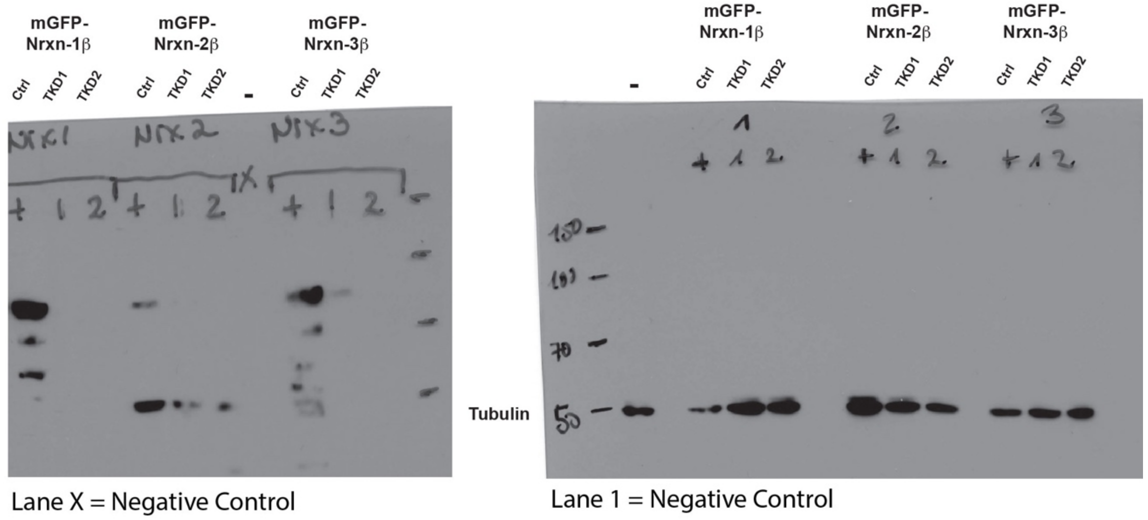

Fig S2. Uncropped blots used in the composition of figure 1. Film to the left shows blot obtained with an antibody raised against EGFP, which was used to tag rat Neurexin-1ß, -2ß, and -3ß. In the Western blot on the right, the same samples were re-probed with an antibody to tubulin as a loading control.
